# New-Onset Mania With Psychotic Features in a Patient With DiGeorge Syndrome, HIV, Syphilis, and Pulmonary Hypertension

**DOI:** 10.7759/cureus.83883

**Published:** 2025-05-11

**Authors:** Gregory Chen, Madeline Pollak

**Affiliations:** 1 Psychiatry, Lehigh Valley Health Network, Allentown, USA

**Keywords:** bipolar, bipolar with psychotic freatures, hiv positive, pulmonary hypertension, syphilis, the 22q11.2 digeorge region

## Abstract

This case report describes a 34-year-old man with HIV and DiGeorge syndrome who experienced his first documented manic episode with psychotic features. On admission, he exhibited psychomotor agitation, catatonic behavior, perseveration, disorganized thoughts, hyper-religiosity, auditory hallucinations, and grandiose delusions. Initial treatment with antipsychotics was complicated by autonomic instability, limiting therapeutic options. Further evaluation revealed syphilis and pulmonary hypertension, which further complicated his hospital course. This case highlights the complexities of managing psychiatric symptoms in patients with DiGeorge syndrome and HIV, as well as the increased risk for first-break psychosis associated with a 22q11.2 deletion.

## Introduction

DiGeorge syndrome (DGS) is thought to result from a microdeletion at 22q11.2 that can cause multiorgan system dysfunction, including cardiac defects, immunodeficiency, hypoparathyroidism, abnormal facies, developmental deficits, and gastrointestinal disorders. The 22q11.2 deletion disrupts genes involved in neurodevelopment, contributing to a heightened risk for psychiatric comorbidities such as schizophrenia and mood disorders [1-4]. Additionally, new-onset psychiatric disorders may present in adolescence or adulthood, and it is important to rule out other organic causes of psychosis, such as infection [5,6]. While anxiety is the most common psychiatric condition, approximately 25% of patients with DGS are diagnosed with schizophrenia [7]. People with 22q11.2 deletion syndrome are significantly more likely than those without the condition to develop schizophrenia, depression, anxiety, and bipolar disorder [2].

Here, we present a complex case of an adult male with a first episode of mania with psychotic features, whose management was complicated by DGS, HIV, syphilis, and pulmonary hypertension [6]. This unique case highlights the role multiple comorbidities, including DGS, play in the management of psychiatric conditions.

## Case presentation

A 34-year-old man with a past medical history significant for HIV and DiGeorge syndrome presented to the hospital following two weeks of erratic behavior, including wandering onto roads, locking himself in his room, and getting into heated arguments with family. Notably, he had received a recent HIV diagnosis in December 2023, which precipitated a dramatic weight loss of 50 lb, leading to hospitalization and subsequent transfer to a rehabilitation facility due to an inability to care for his activities of daily living. He had no prior psychiatric history, trauma, or significant substance abuse, aside from occasional marijuana use and a previous suicide attempt eight years earlier. The patient had been living with his brother and had a diverse work history, including roles as an Uber driver, server, and factory worker, while also navigating a divorce after a five-year separation. He was reported to be compliant at home with his antiretroviral therapy.

Upon admission, the patient exhibited psychomotor agitation and was initially nonverbal, displaying stereotyped movements and perseveration. His initial Bush-Francis Catatonia Rating Scale score was 17. Laboratory evaluations revealed elevated bilirubin (1.3) and protein (8.6) levels, with a CD4 count of 244 (Table 1). A comprehensive drug screen was negative for all substances. Neurologically, he was oriented to self and time but not to location or situation. His thought processes were disorganized, and he demonstrated hyper-religiosity. He reported some auditory hallucinations but denied paranoia or visual hallucinations.

**Table 1:** Laboratory workup that was completed UDS: urine drug screen, RPR: rapid plasma reagin, IM: intramuscular.

Lab	Result	Reference Range	Notes
Total bilirubin	1.3 mg/dL	0.1–1.2 mg/dL	Slightly elevated
Total protein	8.6 g/dL	6.0–8.3 g/dL	Slightly elevated
CD4 count	244 cells/mm³	500–1500 cells/mm³	Significantly low (immunocompromised)
Drug screen (UDS)	Negative	Negative	Normal
RPR (syphilis test)	Positive	Negative	Treated with IM penicillin

When psychiatry was first consulted, the patient was started on Ativan for catatonia and Zyprexa for mood instability and psychosis. However, Zyprexa was later discontinued due to signs of autonomic instability, such as tachycardia and hypertension. During his hospital stay, he exhibited delusions of grandiosity. He claimed he had two children, one born on "June 25," and expressed plans for a new home, car, and phone. He also demonstrated odd visual perceptions, referring to a tree as a "mountain" and claiming to see "angels" in the clouds. His sleep patterns were severely disrupted, with a reduced need for sleep; he often napped throughout the day and slept only 1-2 hours at night.

The patient’s psychiatric evaluation further revealed poor attention, distractibility, hyperactivity, and a tendency for rambling speech, which necessitated frequent redirection to answer questions. His initial treatment focused on stabilizing his mood and addressing the psychotic features. He was started on lithium, which seemed to help reduce his pressured speech and manic symptoms; however, his decreased need for sleep persisted. He was started on Trazodone nightly in an effort to improve his sleep-wake cycle. Despite receiving IM penicillin for a positive RPR indicating syphilis, there was no improvement in his psychosis after the antibiotic course was completed. His delusions, particularly those involving his “daughter,” remained fixed throughout his hospitalization, raising concerns about a potential diagnosis of a schizophrenia spectrum disorder secondary to DiGeorge syndrome. The patient’s family confirmed a diagnosis of DiGeorge syndrome via genetic testing during childhood. His clinical features of DiGeorge syndrome included small auricles, palatal abnormalities, a small mouth, and intellectual disability, per family report.

**Figure 1:**
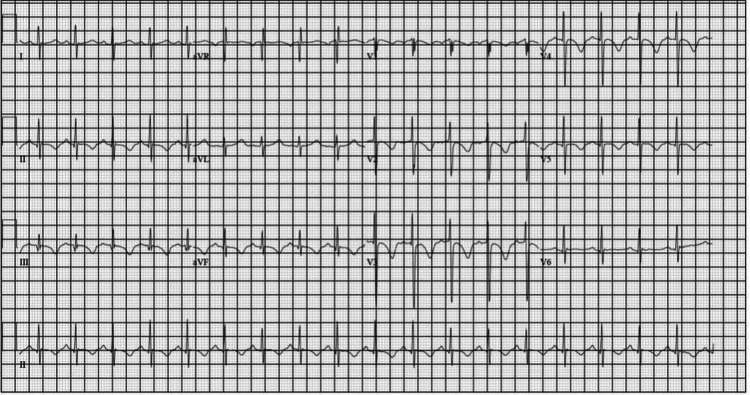
EKG showing T wave abnormalities in anterolateral and inferior leads with a QTc of 514

During his hospital course, it was noted on physical examination that the patient began to exhibit labored breathing. An EKG showed sinus tachycardia with T-wave inversions and a prolonged QTc interval of 514 ms, leading to the discontinuation of both Zyprexa and Trazodone (Figure 1). The patient was then switched to Abilify to target his psychosis, as it is known to have little to no effect on the QTc interval. He responded well to Abilify and showed improvement in mood symptoms and psychosis.

**Figure 2:**
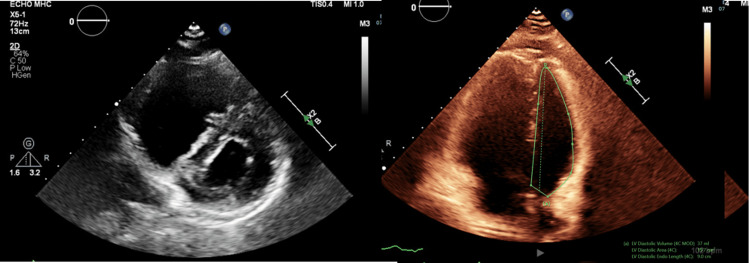
Echocardiographic findings indicate right ventricular dilatation and pulmonary hypertension

Further cardiac workup, including an echocardiogram (Figure 2), revealed right ventricular dilation and pulmonary hypertension, which was confirmed by right heart catheterization showing significantly elevated pulmonary artery pressures. The Pulmonary and Cardiovascular teams were consulted and concluded that his pulmonary hypertension was most likely secondary to his HIV diagnosis.

In summary, the patient was a 34-year-old man with a past medical history of HIV and DiGeorge syndrome. He had no prior psychiatric history, and this was his first documented manic episode with psychotic features. The patient also had no pertinent family history of psychiatric illness.

## Discussion

The most common variant of DGS interferes with 46 protein-encoding genes [1]. The dysfunction of these genes causes the clinical presentation of DGS. One of the deleted protein-encoding genes is the COMT gene, which encodes the enzyme catechol-O-methyltransferase. This enzyme regulates dopamine metabolism, both centrally and peripherally [2]. The deletion of the COMT gene and the subsequent increase in dopamine are thought to be responsible for the link between DGS and psychiatric disorders, especially schizophrenia [5]. Although no direct association has been found between DGS and mood disorders, the deletion of the COMT gene likely influenced the presence of psychotic features seen in our patient [1,3,4]. Due to the high prevalence of schizophrenia, patients with DGS are often prescribed antipsychotics. In our case, the patient experienced adverse cardiac effects and autonomic instability, most likely due to Zyprexa and Trazodone. There is significant overlap in the affected systems, particularly cardiac and neurologic, and this comorbidity can greatly complicate management, as demonstrated in our case [3].

Additionally, the patient remained compliant with antiretroviral therapy prior to admission, and his CD4 count remained stable, making HIV-associated delirium less likely in the differential. His psychosis and mental status also did not improve after completing treatment for syphilis, ruling out neurosyphilis as the etiology of his psychosis [6]. While both HIV- and syphilis-associated delirium can present with psychiatric symptoms, they are typically more acute and reversible with appropriate treatment.

Due to the variation in clinical presentations, DGS patients should receive a multidisciplinary approach to management, including a personalized combination of physicians and therapists. This may involve geneticists, immunologists, physical/speech/occupational therapists, cardiologists, endocrinologists, nephrologists, gastroenterologists, and psychiatrists. Given our patient’s comorbidities, he required follow-up with psychiatry, infectious disease, and pulmonology. However, long-term follow-up data were unavailable, limiting our ability to assess sustained treatment outcomes. Additionally, findings from this single case may not be generalizable to broader patient populations with differing comorbidity profiles.

## Conclusions

The medical comorbidities in this patient underscore the complexity of treating a first episode of mania with psychotic features. This patient had multiple predisposing biological factors, including DiGeorge syndrome, syphilis, HIV, and pulmonary hypertension. In addition, he had metabolic and hematologic abnormalities that made pharmacotherapy more challenging. Despite appropriate pharmacological interventions, the patient’s management was complicated by autonomic instability and the persistence of his mania and delusions. Future research should consider the unique medical complications associated with 22q11.2 deletion syndrome and explore the use of antipsychotics such as aripiprazole, brexpiprazole, or cariprazine. These agents may offer effective management of mood and psychotic symptoms while potentially reducing the risk of cardiac complications commonly observed in this population. Furthermore, the medical and psychiatric nuances in this case highlight the importance of interdisciplinary collaboration in treating a medically and psychiatrically complex patient.
